# Supplementary material for: The Impact of Dietary Factors during Pregnancy on the Development of Islet Autoimmunity and Type 1 Diabetes: A Systematic Literature Review
Source: Nutrients. 2023 Oct 11;15(20):4333. doi: 10.3390/nu15204333 (PMC10609322; doi:10.3390/nu15204333)
Supplement: Supplementary file 1 [file nutrients-15-04333-s001.zip › Supplemental Material Figure S1.pdf]

PRISMA 2020 flow diagram for updated systematic reviews which included searches of databases, registers and other sources

Previous studies

Identification

Studies included in previous version of review (n = 0)

Reports of studies included in previous version of review (n = 0)

Identification of new studies via databases and registers

Records identified from*:
- Databases (n = 343)
- Registers (n = 0)

Records removed *before screening*:
- Duplicate records removed (n = 99)
- Records marked as ineligible by automation tools (n = 0)
- Records removed for other reasons (n = 0)

Screening

Records screened (n = 244)

Records excluded** (n = 0)

Reports sought for retrieval (n = 244)

Reports not retrieved (n = 2)

Reports assessed for eligibility (n = 242)

Reports excluded: 219
- Maternal diabetes (n = 41)
- Irrelevant exposure (n = 47)
- Irrelevant study design (n = 38)
- Irrelevant outcome (n = 26)
- Irrelevant review (n = 64)
- Textbook (n = 1)
- Protocol for future study (n = 1)
- Protocol for study already included (n = 1)

Included

New studies included in review (n = 35)
Reports of new included studies (n = 35)

Total studies included in review (n = 35)
Reports of total included studies (n = 35)

Identification of new studies via other methods

Records identified from:
- Websites (n = 0)
- Organisations (n = 0)
- Citation searching (n = 20)
- etc.

Reports sought for retrieval (n = 20)

Reports not retrieved (n = 0)

Reports assessed for eligibility (n = 20)

Reports excluded: 8
- Meta analysis (n =1)
- Irrelevant exposure (n = 4)
- Irrelevant study design (n = 1)
- Article only found as citation (n = 2)

*Consider, if feasible to do so, reporting the number of records identified from each database or register searched (rather than the total number across all databases/registers).
**If automation tools were used, indicate how many records were excluded by a human and how many were excluded by automation tools.

From: Page MJ, McKenzie JE, Bossuyt PM, Boutron I, Hoffmann TC, Mulrow CD, et al. The PRISMA 2020 statement: an updated guideline for reporting systematic reviews. BMJ 2021;372:n71. doi: 10.1136/bmj.n71. For more information, visit: http://www.prisma-statement.org/
