# Supplementary material for: The Impact of Dietary Factors during Pregnancy on the Development of Islet Autoimmunity and Type 1 Diabetes: A Systematic Literature Review
Source: Nutrients. 2023 Oct 11;15(20):4333. doi: 10.3390/nu15204333 (PMC10609322; doi:10.3390/nu15204333)
Supplement: Supplementary file 1 [file nutrients-15-04333-s001.zip › Supplemental Material Table S1.pdf]

Table S1

**Data base**	**Advanced search string**
**Scopus**	TITLE-ABS-KEY (("type 1 diabetes" OR "type I diabetes") AND "prenatal" AND (diet OR "islet autoimmunity" OR "animal*" OR "mouse" OR "mice" OR "nutrient" OR "gluten" OR "gliadin" OR "vitamins" OR "milk" OR "fibres")) AND (LIMIT-TO (LANGUAGE , "English"))
**PubMed**	TITLE-ABS ((type 1 diabetes OR type I diabetes) AND prenatal AND (diet OR "islet autoimmunity" OR animal* OR mouse OR mice OR nutrient OR gluten OR gliadin OR vitamins OR milk OR fibres))
